# Syngeneic tobacco carcinogen–induced mouse lung adenocarcinoma model exhibits PD-L1 expression and high tumor mutational burden

**DOI:** 10.1172/jci.insight.145307

**Published:** 2021-02-08

**Authors:** Laura P. Stabile, Vinod Kumar, Autumn Gaither-Davis, Eric H. Huang, Frank P. Vendetti, Princey Devadassan, Sanja Dacic, Riyue Bao, Richard A. Steinman, Timothy F. Burns, Christopher J. Bakkenist

**Affiliations:** 1 Department of Pharmacology and Chemical Biology, University of Pittsburgh School of Medicine, Pittsburgh, Pennsylvania, USA.; 2 UPMC Hillman Cancer Center, Pittsburgh, Pennsylvania, USA.; 3 Division of Hematology/Oncology, Department of Medicine;; 4 Department of Radiation Oncology; and; 5 Department of Pathology, University of Pittsburgh School of Medicine, Pittsburgh, Pennsylvania, USA.

**Keywords:** Oncology, Lung cancer, Mouse models

## Abstract

Human lung adenocarcinoma (LUAD) in current or former smokers exhibits a high tumor mutational burden (TMB) and distinct mutational signatures. Syngeneic mouse models of clinically relevant smoking-related LUAD are lacking. We established and characterized a tobacco-associated, transplantable murine LUAD cell line, designated FVBW-17, from a LUAD induced by the tobacco carcinogen 4-(methylnitrosoamino)-1-(3-pyridyl)-1-butanone in the FVB/N mouse strain. Whole-exome sequencing of FVBW-17 cells identified tobacco-associated *Kras^G12D^* and *Trp53* mutations and a similar mutation profile to that of classic alkylating agents with a TMB greater than 500. FVBW-17 cells transplanted subcutaneously, via tail vein, and orthotopically generated tumors that were histologically similar to human LUAD in FVB/N mice. FVBW-17 tumors expressed programmed death ligand 1 (PD-L1), were infiltrated with CD8^+^ T cells, and were responsive to anti–PD-L1 therapy. FVBW-17 cells were also engineered to express green fluorescent protein and luciferase to facilitate detection and quantification of tumor growth. Distant metastases to lung, spleen, liver, and kidney were observed from subcutaneously transplanted tumors. This potentially novel cell line is a robust representation of human smoking-related LUAD biology and provides a much needed preclinical model in which to test promising new agents and combinations, including immune-based therapies.

## Abstract 2

We established and characterized a clinically relevant tobacco carcinogen-induced lung adeno-carcinoma syngeneic cell line that will be useful for preclinical evaluation of immune-oncology agents.

## Introduction

The therapeutic landscape for non–small cell lung cancer (NSCLC) has rapidly transformed based on histologic subtype, molecular profile, and immunophenotype. In the majority of patients, there are no targetable oncogenic drivers. Therefore the field of immuno-oncology has rapidly evolved for NSCLC, and immune-based therapies, including immune checkpoint inhibitors (ICIs) such as antibodies against programmed death 1 (PD-1) and programmed death ligand 1 (PD-L1), have emerged as exciting therapeutic approaches (1). However, only approximately 20% of patients with advanced NSCLC respond clinically to current ICIs (2, 3). While high tumor mutational burden (TMB), a characteristic of smoking-related cancers, and tumoral PD-L1 expression are the most well-validated predictive biomarkers for response to ICIs across multiple cancer types (4–6), preclinical autochthonous murine models of NSCLC are urgently needed to serve as a platform for novel immune-related biological insights and testing therapeutic strategies.

Our ability to understand changes in the lung tumor microenvironment (TME), especially the immune environment, is limited by the lack of appropriate preclinical models. The only robust and widely used syngeneic lung cancer model, the Lewis lung carcinoma (LLC) model (7), was derived from a spontaneous lung carcinoma in a C57BL/6 mouse. The LLC model does not recapitulate exposure to tobacco carcinogen, which is the principal driver of lung cancer in patients as well as the primary mutagen that generates tumor neo-antigens that may be key to response to ICIs. The CMT64 model, also established from a spontaneous alveolar lung carcinoma in a C57BL/6 mouse, and its metastatic sublines also have limited utility because they again do not represent exposure to tobacco carcinogen (8). LM2 cells, derived from a urethane-induced lung tumor in A/J mice, are not widely used, and the molecular features and biological phenotypes are not well characterized (9, 10). Similarly, while genetically engineered mouse models (GEMMs) of lung cancer are good for the evaluation of oncogenic drivers and targeted therapies, they do not represent clinical lung tumorigenesis and lack mutational diversity (11).

Among the tobacco carcinogenic compounds, nicotine-derived nitrosamines, such as 4-(methylnitrosoamino)-1-(3-pyridyl)-1-butanone (NNK), and its metabolites, are the most abundant and the strongest carcinogens (12, 13). The FVB/N mouse does not develop spontaneous lung tumors, but it is susceptible to chemically induced lung carcinomas (11) and a commonly used strain for generation of GEMMs (14–16). We previously characterized the development of NNK-induced lung adenocarcinoma (LUAD) in FVB/N mice, where pulmonary preneoplasias were found as early as 4 weeks post-NNK (17). These hyperplastic lesions, found in the small airways and the lung parenchyma, consist of tightly packed epithelial cells displaying a papillary growth pattern and decreased nuclear-to-cytoplasmic ratio (18). They are histologically similar to the human preneoplastic lesion and LUAD precursor, atypical adenomatous hyperplasia, often found in smokers (19), which progresses to *KRAS*-mutant LUAD over time (18, 20). While gene expression signatures in NNK-induced lung tumors are highly comparable to those found in human LUAD (21), our knowledge of the murine mutational landscape, such as the smoking-associated mutational patterns characterized in human LUAD (22), is limited. The long latency time for full LUAD development in the NNK model, lack of metastatic potential, and labor-intensive quantification of complete lung tumor burden that can only be performed postmortem restrict the model’s usefulness for routine preclinical therapeutic testing.

To overcome these challenges, we developed and characterized a clinically relevant tobacco carcinogen–induced (NNK-induced) LUAD syngeneic cell line to facilitate preclinical testing of potential therapeutic approaches in an immune-competent environment. FVBW-17 is the first tobacco carcinogen–induced murine lung tumor cell line, we believe, that represents human LUAD disease. To our knowledge, this is the only syngeneic model derived from a lung tumor induced by a tobacco carcinogen that is easily transplantable in FVB/N mice, grows reproducibly, and metastasizes to multiple sites. This tobacco carcinogen–induced syngeneic model of LUAD with PD-L1 expression, high TMB, and responsiveness to anti–PD-L1 therapy is a critical technical advance that will allow impactful preclinical experimental studies.

## Results

### Establishment and characterization of FVBW-17 cell line.

**Figure 1:**
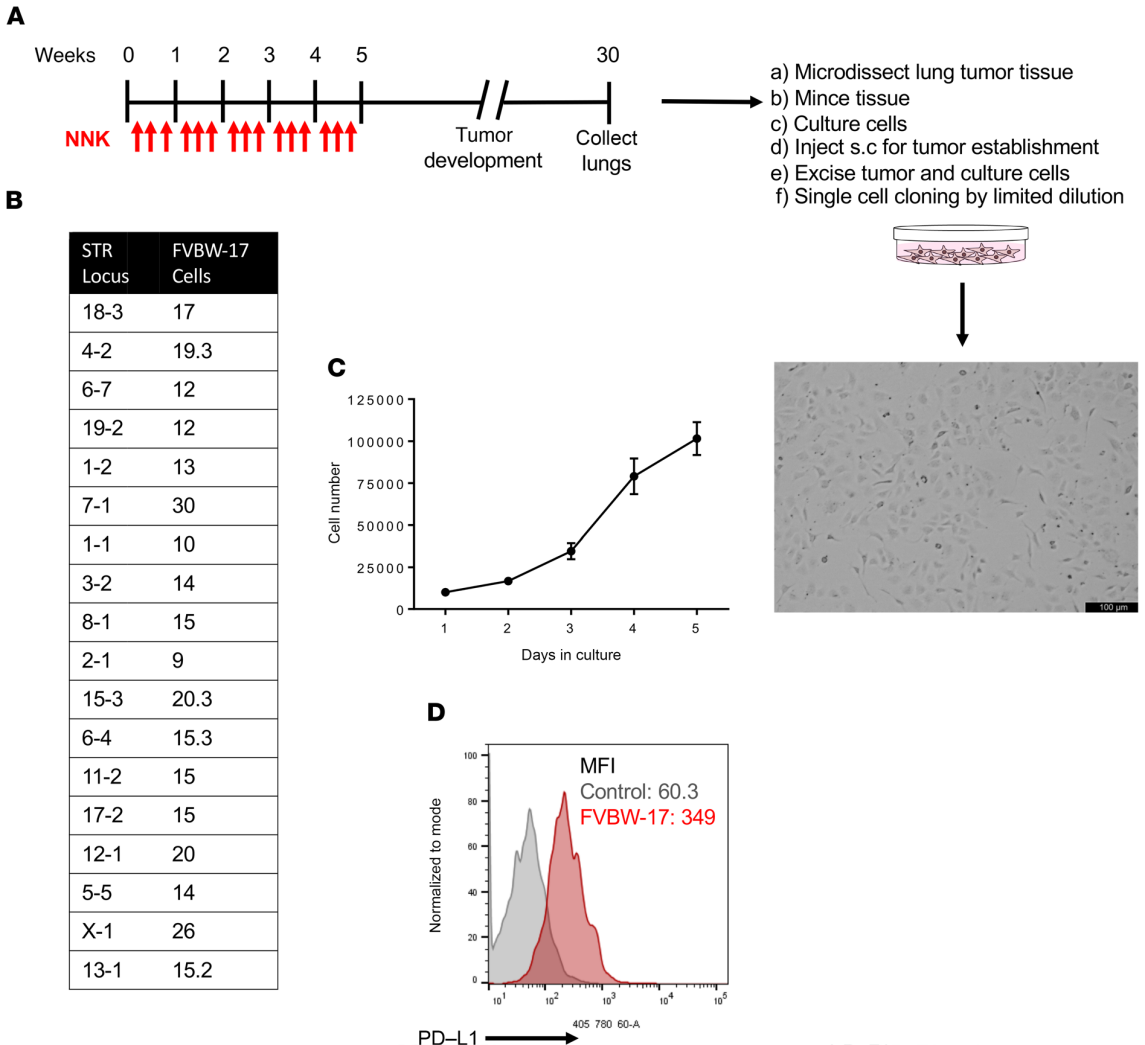
Generation and characterization of the murine FVBW-17 cell line. (**A**) Schema of the establishment of the parental FVBW-17 cell line and clonal lines and representative phase-contrast microscopic image of FVBW-17 cells in culture. Scale bar: 100 μm. (**B**) STR profile of the FVBW-17 cell line. (**C**) Cell growth properties of FVBW-17 cells in culture. Results are reported as the mean ± SD of 6 replicates. (**D**) Flow cytometry for PD-L1 expression of cultured cells. Results are expressed as mean fluorescence intensity, which is a measure of protein expression compared with the control (fluorescence minus one, which is viability dye without PD-L1 antibody).

We have previously described the use of the NNK-induced lung cancer model for both preventive and treatment therapies, and this model has been used extensively to study NSCLC initiation and progression (14, 17, 18, 20, 23). However, this model has a long latency period for adenocarcinoma and lacks metastatic potential. In order to improve the utility of the model for preclinical testing, we treated mice with NNK and isolated primary tumors to establish a syngeneic NNK-induced mouse lung cancer cell line as shown in the schema in Figure 1A. A parental line and 3 clonal lines were established. A DNA sample from the parental and clonal lines developed was submitted to ATCC for characterization and authentication using its mouse short tandem repeat (STR) profiling service (Figure 1B). These cells exhibit typical NSCLC morphology under light microscopy and a rapid doubling time in culture of 35 hours (Figure 1C). In order to support the use of this line for studies evaluating antibodies targeting the PD-1/PD-L1 axis, in vitro PD-L1 expression in FVBW-17 cells was examined by flow cytometry. We observed robust basal expression of PD-L1, 6-fold higher than the control (Figure 1D), which was confirmed by Western analysis (not shown).

### FVBW-17 cell lines display a prominent alkylating molecular signature and genetic features of human LUAD.

**Figure 2:**
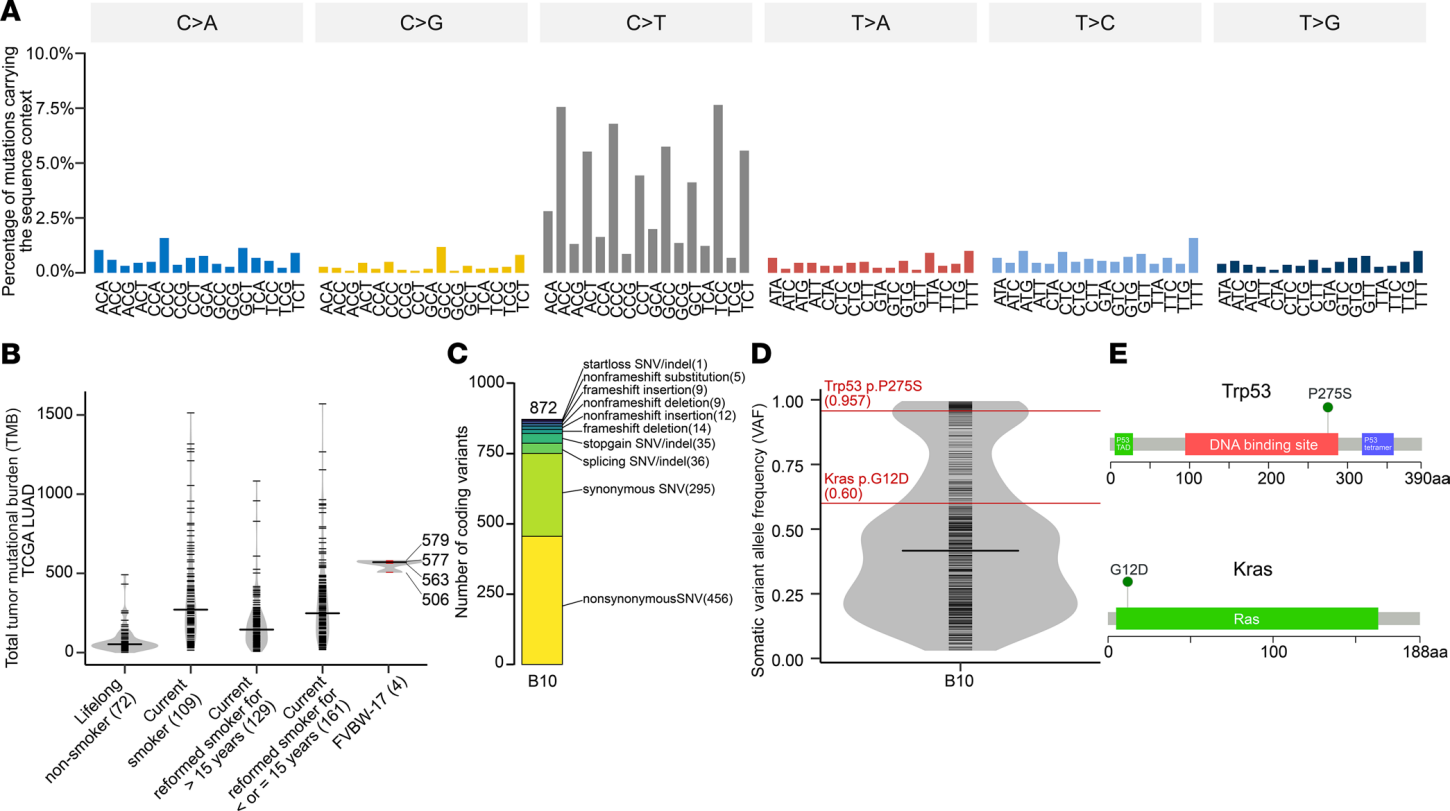
Somatic mutation profile of FVBW-17 represents a classic alkylating signature with validated *Kras* and *Trp53* mutations. (**A**) Pattern of the FVBW-17 mutation signature associated with NNK using a 96-substitution classification. Variants were grouped by the trinucleotide sequence context centered at the mutated base shown on the *x* axis. The percentage of variants carrying the trinucleotide sequence context out of the whole mouse genome is shown on the *y* axis. (**B**) Comparison of FVBW-17 TMB to that of primary tumors from TCGA LUAD patients grouped by tobacco smoking history. Each short red horizontal line indicates the TMB value of each FVBW-17 line (median = 570). The numbers on the right show the TMBs for each of the 4 lines. TMB was defined as the total number of somatic mutations predicted to affect protein sequences (non-synonymous somatic mutations, NSSMs). *n* = 471 samples shown. Each shorter black horizontal line represents 1 tumor. Longer black horizontal line represents median of TMB in each group. (**C**) Distribution of different variant types that affect the coding regions of the genome. Ten categories are shown based on exonic function annotation of each variant (synonymous mutations included), with numbers from each category shown in between parentheses. (**D**) Distribution of somatic VAFs across coding variants from **C**. The VAFs of *Trp53* P275S and *Kras* G12D are shown as red horizontal lines. *n* = 872 coding variants shown. Each shorter black horizontal line represents 1 variant. Longer black horizontal line represents median of VAF. (**E**) Lollipop plot showing the location of *Trp53* and *Kras* somatic mutations in the protein domain. Trp53 green box: transactivation domain. Trp53 red box: DNA binding domain. Trp53 blue box: tetramerization domain. Kras green box: Ras domain.

We performed whole-exome sequencing (WES) of FVBW-17 cell lines to evaluate the similarity to human LUAD. Signatures of mutational processes attributed to known mutagenic exposures have been previously reported (24). We analyzed the sequence context–specific mutation signature of FVBW-17 cells, which revealed a striking similarity to human cancer Signature 11, with a low-high, low-high repetitive pattern within the C>T group (Figure 2A and Supplemental Figure 1; supplemental material available online with this article; https://doi.org/10.1172/jci.insight.145307DS1), which does not preferentially occur at CpG sites from spontaneous deamination of methyl-cytosine, leading to the age-related Signature 1 (24). Signature 11 has the mutational pattern reported in experimental studies of alkylating agents (25). NNK can be activated to a pyridyloxobutylating agent, and this alkylating agent creates O6-methylguanine adducts, which lead to G>A mutations (C>T on the opposite strand) (26). We next calculated the total TMB, defined as the total number of nonsynonymous mutations from the whole exome in the 4 FVBW-17 cell lines, and found a median TMB of 570 per exome (range 506–579), approximately 2-fold higher than the median TMB found in current and former smokers using the The Cancer Genome Atlas (TCGA) human LUAD cohort (Figure 2B). Figure 2C depicts the number of coding variants identified by type. Since *KRAS* and *TP53* are mutated at high frequencies in human LUAD, occur concurrently in many cases (27), and are typically found in tumors from patients who smoke (28, 29), we specifically focused on identifying these mutations. We found a high variant allele frequency (VAF) of both *Trp53* (P275S; 0.957) and *Kras* (G12D; 0.60) (Figure 2D). While *Trp53* P275S (equivalent to human P278S) is not a common mutation, it is located in the DNA binding domain, which is a mutational hot spot. Figure 2E shows the location of the *Trp53* and *Kras* mutations in the protein domains. *Kras^G12D^* was confirmed by Sanger sequencing, and p53 protein expression (a surrogate that correlates with p53 hot spot mutations) was confirmed by Western blot analysis (data not shown). No other oncogenic activating mutations were found.

### FVBW-17 cells grow rapidly in syngeneic FVB/N mice, and tumors maintain a similar histomorphology and immunophenotype to human LUAD.

**Figure 3:**
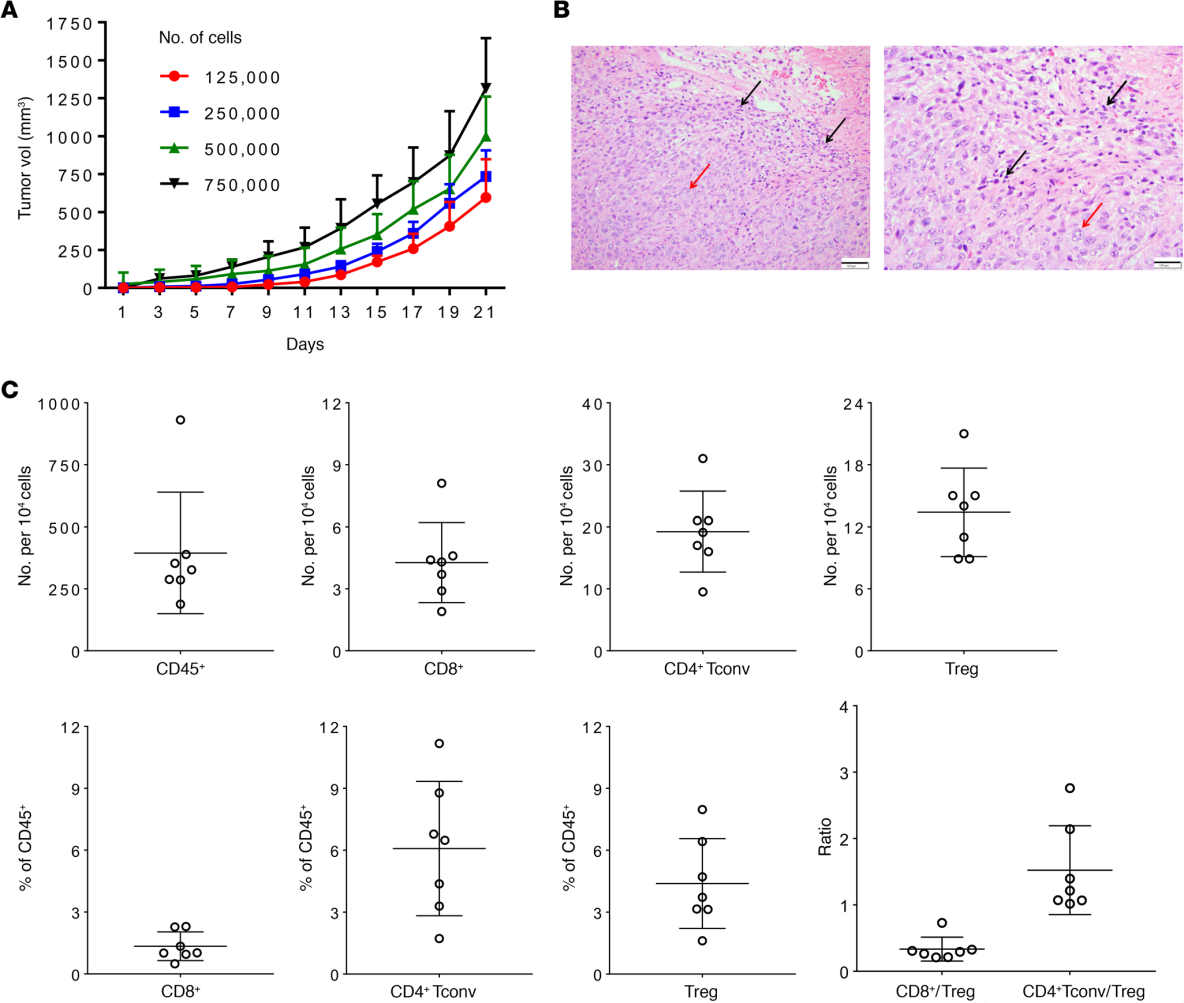
FVBW-17 cells grow as subcutaneous tumors in FVB/N mice, are histologically similar to human LUAD, and contain tumor-infiltrating lymphocytes. (**A**) A total of 1.25 × 10^5^ to 7.5 × 10^5^ FVBW-17 cells were injected s.c. into hind flanks in FVB/N mice (*n* = 8–10 mice per cell density). Tumors were measured every 2 days for 21 days. Results represent the mean ± SD. (**B**) Representative H&E images (4×, left panel; 20×, right panel) of s.c. FVBW-17 tumor showing malignant tumor with morphologic features similar to human LUAD. Black arrows, representative tumor-infiltrating lymphocytes; red arrow, representative tumor cell. Scale bar: 100 μm. (**C**) Immunophenotyping of FVBW-17 cells grown as s.c. tumors collected at day 21 (*n* = 7 mice). Each cell type is expressed as number per 10^4^ cells (upper panel) or percentage of CD45^+^ cells (lower panel). The ratio of CD8^+^/Treg and CD4^+^/Treg is also shown. Results are expressed as the mean ± SD.

To evaluate the tumorigenic potential of FVBW-17 cells, we injected 1.25 × 10^5^ to 7.5 × 10^5^ cells subcutaneously (s.c.) in the hind flank and monitored tumor growth over 21 days. As shown in Figure 3A, FVBW-17 cells resulted in rapid tumor growth that was proportional to the number of cells injected. By 4 weeks, tumors were aggressive and exceeded 10% of the body weight in the 5 × 10^5^ and 7.5 × 10^5^ groups. All 3 clonal lines grew at a similar rate (data not shown). Tumors were removed, fixed, and paraffin embedded for histological evaluation. H&E-stained sections demonstrated malignant tumor with morphological features similar to LUAD in humans (Figure 3B). Similar to human LUAD, the tumor was composed of sheets and nests of large polygonal cells with vesicular nuclei, occasional prominent nucleoli, and moderate amounts of cytoplasm (Figure 3B, red arrows). Tumor nests were surrounded by fibromyxoid desmoplastic stroma with scattered lymphocytes (Figure 3B, black arrows). The tumor was poorly differentiated because no morphological features of glandular, squamous, or neuroendocrine differentiation could be found on the routine H&E sections at least as an s.c. model.

To determine the extent of immune infiltration in s.c. tumors, we performed immune profiling of CD45^+^ cells in a subset of mice at day 21 (Figure 3C). CD8^+^ T cells, conventional CD4^+^ T cells, and Tregs were quantified as total number per 10^4^ cells as well as a percentage of CD45^+^ immune cells. We found that T cell populations made up less than 10% of the total CD45^+^ immune cells and exhibited low CD8^+^ and CD4^+^ T cell/Treg ratios. In general, these results suggest that FVBW-17 tumors were weakly infiltrated with CD8^+^ T cells. However, these values were similar to what we have previously observed in the syngeneic CT26 colon cancer model (30). The relatively low T cell infiltrate that we observed may be due to the late time point of tumor extraction and the presence of necrosis in some tumors.

**Figure 4:**
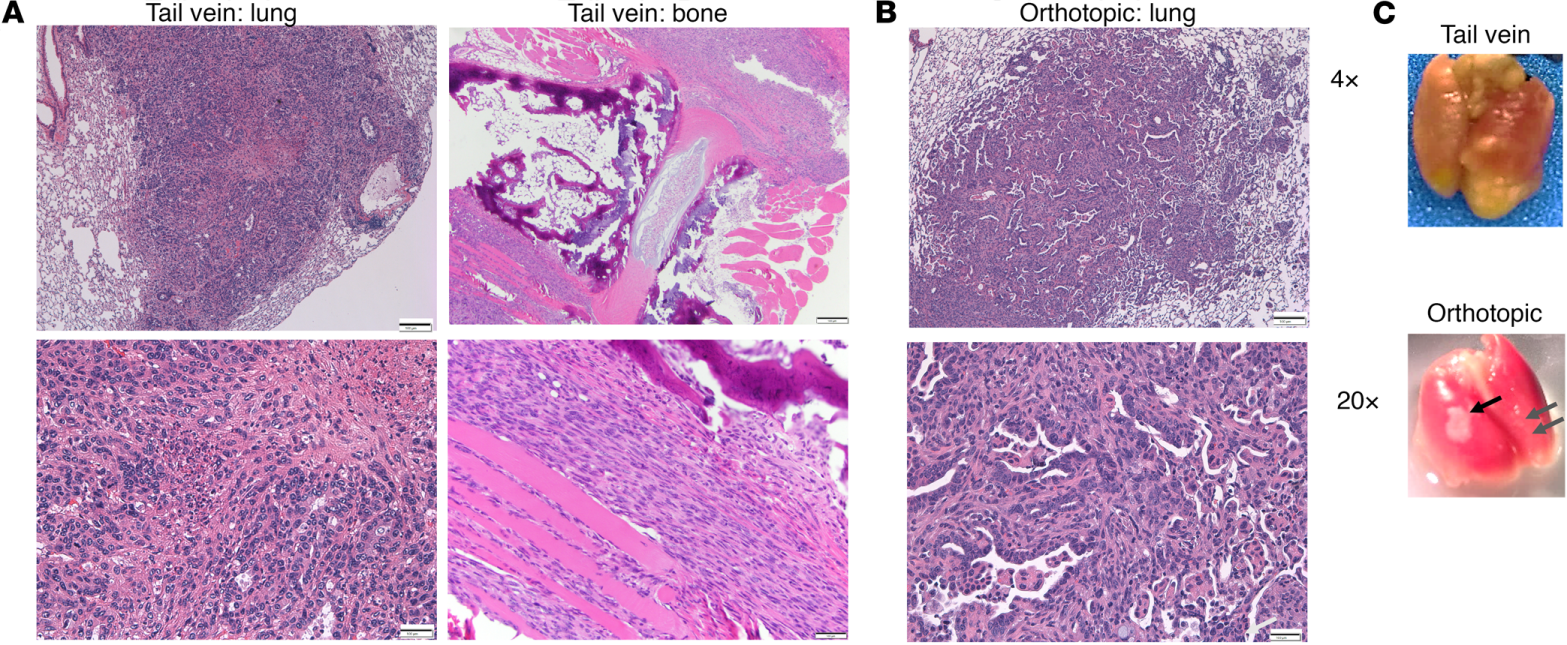
FVBW-17 cells seed the lung following i.v. injection and form lung nodules by direct orthotopic intrathoracic injection. (**A**) FVBW-17 cells (5 × 10^5^) were injected into the lateral tail vein. Lungs were formalin-inflated 21 days postinjection for evidence of lung tumor burden by H&E, showing NSCLC with rare glandular structures and single cells invading the stroma and necrosis (left panels). Right panels show bone metastasis. Scale bar: 100 μm. (**B**) FVBW-17 tumor establishment in the lung 15 days after direct intrathoracic injection of 1 × 10^6^ cells in PBS/1.35 mg/mL Matrigel. H&E shows evidence of invasive adenocarcinoma with malignant glandular structures. Scale bar: 100 μm. (**C**) Gross lung from tail vein model (top panel) or orthotopic model (bottom panel). Black arrow shows primary tumor; gray arrows show lung metastasis.

To determine if FVBW-17 cells could serve as a platform to understand lung metastasis and tumor growth within the lung environment, we evaluated whether FVBW-17 cells could colonize the lung following lateral tail vein injection or through direct implantation in the lungs. Ten mice (5 male/5 female) were injected intravenously (i.v.) with 1 × 10^6^ FVBW-17 cells. After 4–5 weeks, mice were sacrificed, and lungs and other tissues were collected and fixed for histologic evaluation. We found metastatic lung nodules in all mice as shown in H&E stains (Figure 4A) and in gross lungs (Figure 4C). In 1 mouse, we also observed bone metastases but no evidence of tumor growth in any other tissues at this time point (Figure 4A). To evaluate primary tumor growth within the lung microenvironment, we transplanted 1 × 10^6^ FVBW-17 cells orthotopically to the lungs of 22 FVB/N mice (12 female/10 male), which led to the formation of well-differentiated lung adenocarcinomas in 100% of the mice with a median primary tumor size of 1.31 mm^3^ (range: 0.49–2.72 mm^3^) as assessed by H&E-stained sections (Figure 4B) and gross lungs (Figure 4C, black arrow). By 2 weeks postimplantation, mice had difficulty breathing due to primary tumor burden. Although we did not observe metastasis to distant tissues with this model, we did observe spread to the contralateral lobe of the lung in 5/22 mice (Figure 4C, gray arrows).

### Establishment of cell lines that stably express GFP and luciferase.

**Figure 5:**
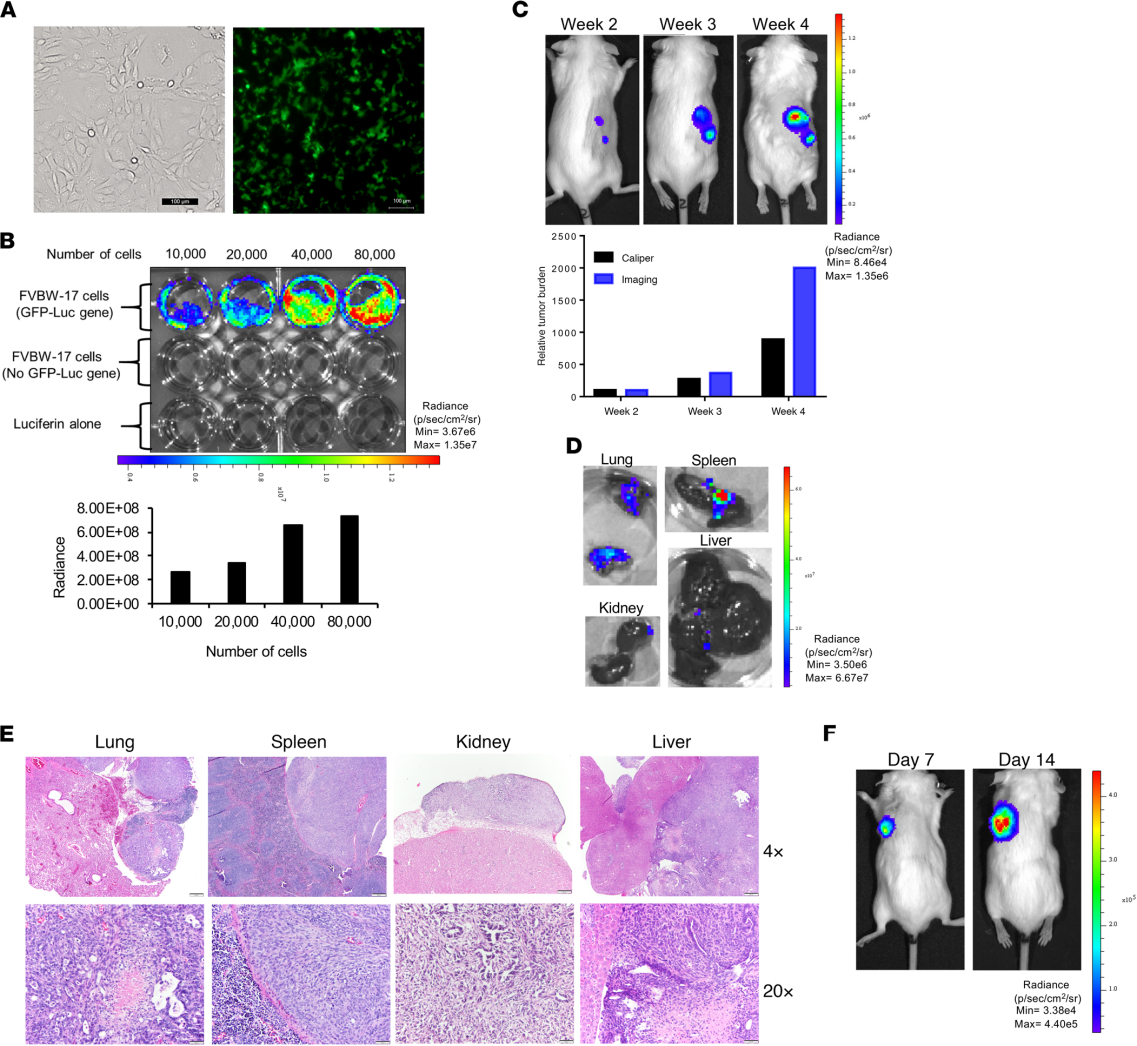
Bioluminescence imaging facilitates detection of subcutaneous tumor growth and metastasis. (**A**) FVBW-17 cells transduced with lentivirus for GFP-Luc showed high transduction efficiency by comparison with phase-contrast (left) and fluorescence microscopy (right). Scale bar: 100 μm. (**B**) Luciferase assay of GFP-Luc cells and control cells with varying cell number and quantification of luciferase assay (radiance). (**C**) Subcutaneous injection of FVBW-17 GFP-Luc cells (2.5 × 10^5^) and tumor growth over 4 weeks monitored in vivo by IVIS imaging. (**D**) Ex vivo bioluminescence imaging of various organs was used to identify metastatic spread in lung, spleen, kidney, and liver. (**E**) Sites of metastases found on IVIS imaging from subcutaneous FVBW-17 tumors confirmed by H&E histology (original magnification, 4×, top panel; 20×, bottom panel). Scale bar: 100 μm. (**F**) Imaging of orthotopically injected cells.

FVBW-17 cells were stably transfected with a lentiviral construct conferring expression of GFP and firefly luciferase (GFP-Luc), allowing tumor growth to be monitored in real time using in vivo bioluminescence imaging. The GFP-positive FVBW-17 cells were sorted into high and low GFP-positive cells by FACS. The high GFP-positive cells were expanded for use in all in vivo experiments. Figure 5A shows high GFP expression by comparing the phase-contrast image with the fluorescence image. We also detected a cell number–dependent increase in luciferase expression on cells grown in vitro (Figure 5B). We found that FVBW-17 GFP-Luc cells implanted s.c. into FVB/N mice formed rapidly growing tumors that could be imaged and quantified over time and showed a slightly higher luminescence signal as compared with caliper measurements due to the capability of 3D tomography in imaging (Figure 5C). The s.c. tumors were surgically excised, and mice were monitored for distant metastases up to 2 additional months. In mice euthanized at 1 month or longer after primary tumor removal, we detected metastases to lung, spleen, kidney, and liver as detected by ex vivo imaging (Figure 5D) and histologic confirmation (Figure 5E). H&E sections of metastases showed invasive adenocarcinoma composed of malignant glands and solid sheets of epithelioid cells. Supplemental Table 1 shows the frequency of metastases to these tissues. Central necrosis was also present in some cases. To confirm that the GFP-Luc cells could also grow within the lung and be imaged, we also injected these cells orthotopically. In vivo imaging detected tumor growth in the lung over time (Figure 5F).

### Effect of PD-L1 inhibition on FVBW-17 tumor growth in vivo.

**Figure 6:**
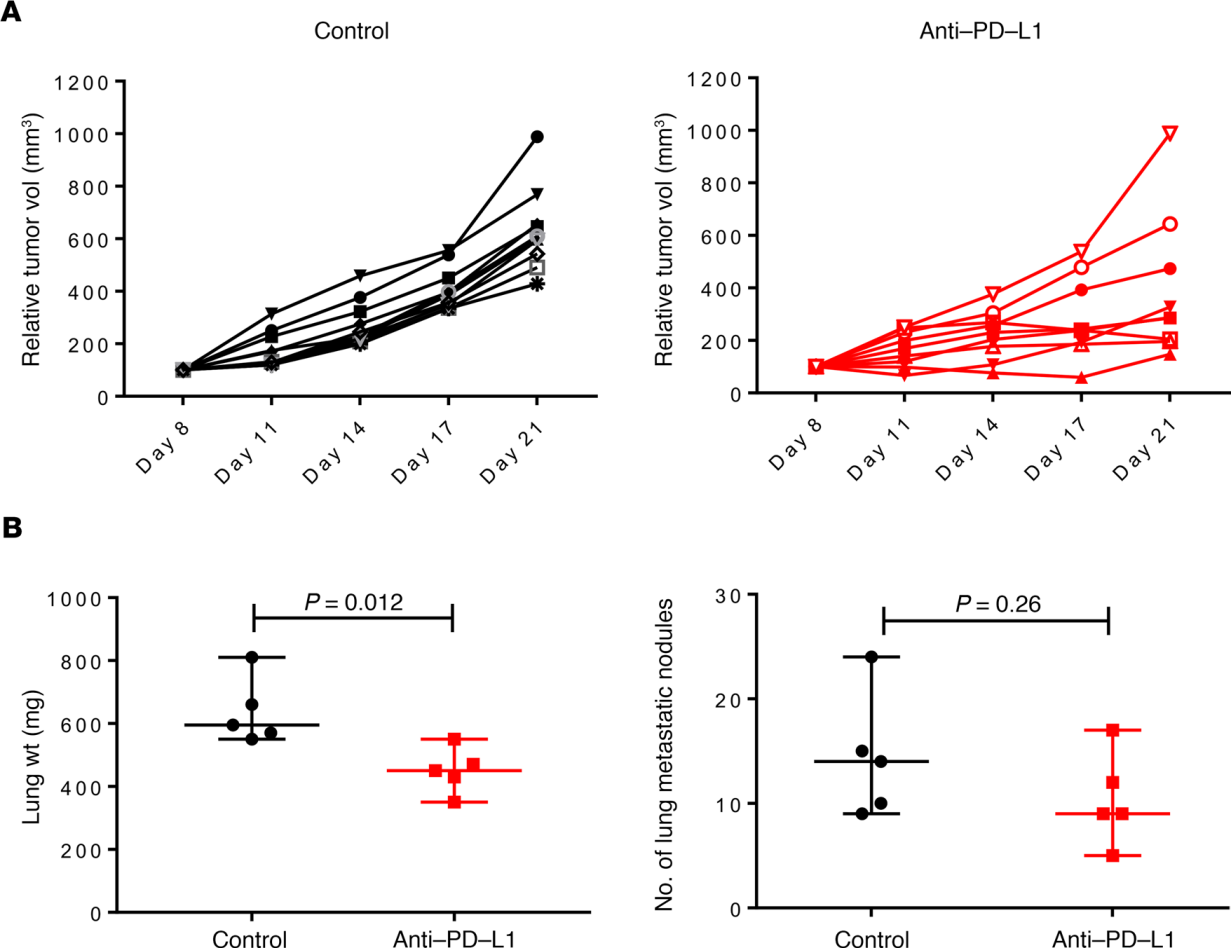
Growth kinetics of FVBW-17 cells treated with anti–PD-L1 in vivo. (**A**) A total of 5 × 10^5^ FVBW-17 cells were injected s.c. into FVBW-17 mice (*n* = 8–10 per group) on day 1. Palpable tumors were identified by day 7. Anti–PD-L1 or isotype control antibody (10 mg/kg) was administered 2 times per week for 2 weeks. Tumor measurements were recorded every 3 days and plotted as relative tumor volume for each individual tumor over time. Each line represents an individual tumor. (**B**) A total of 1 × 10^6^ FVBW-17 cells were injected i.v. into FVB/N mice (*n* = 5 mice/group). Anti–PD-L1 or isotype control (10 mg/kg) was administered 1 week following tumor injection and continued 2 times per week for 3 weeks. Both lung weight and number of lung metastatic nodules were evaluated. Data shown as mean ± SD. *P* values calculated using Student’s *t* test.

To determine the therapeutic effect of PD-L1 targeting in this model, FVBW-17 cells were implanted s.c. into FVB/N mice. When tumors were palpable after 1 week, anti–PD-L1 or control treatments commenced with twice weekly dosing for a total of 4 doses (2 weeks) by i.p. injection. Comparison of individual tumor growth in each treatment group demonstrated that a subset of tumors had suppressed growth of FVBW-17 compared with control treatment. Importantly, we found that 6/10 (60%) mice had evidence of lung metastases in the control group, compared with only 2/8 (25%) in the group treated with anti–PD-L1 (Figure 6A). To determine the importance of the lung TME in mediating response to anti–PD-L1, we also tested a 3-week anti–PD-L1 treatment in the lung metastasis model and found a significant decrease in lung weight (*P* = 0.012) and a lower median number of lung metastatic nodules in the group treated with anti–PD-L1 compared with control-treated mice (Figure 6B). These results suggest preliminary anti–PD-L1 activity, providing a potentially new experimental model with robust growth to evaluate novel immunotherapeutic agents.

## Discussion

A major barrier to identifying new treatment options and improving survival in NSCLC is the lack of preclinical models that mimic the human disease, including metastasis and immune-competent models. We have generated and characterized a potentially novel, clinically relevant tobacco carcinogen–induced (NNK-induced) LUAD syngeneic cell line that will facilitate in vivo preclinical testing of targeted and immune therapies for NSCLC. Tobacco smoke is made up of thousands of chemicals and more than 60 carcinogens, including NNK (12). NNK is a heterocyclic nitrosamine with potent lung-carcinogenic properties in both humans and mice (31–33). NNK metabolites have been detected in urine of smokers and those exposed to secondhand tobacco smoke (34), and elevated levels of NNK are associated with lung cancer risk (35). NNK-induced lung carcinogenesis has been well characterized in mice and in other animal models, including rats, hamsters, and ferrets (36). NNK initiates carcinogenesis in part through its metabolic conversion to the electrophilic metabolite 4-(methylnitrosamino)-1-(3-pyridyl)-1-butanol (NNAL), which binds to DNA, forming DNA adducts and in turn causing dysregulated gene replication (32, 36). NNK-derived DNA damage occurs through metabolic activation of NNK and NNAL to DNA alkylating agents, generating pyridylhydroxybutyl DNA adducts and methyl adducts, respectively (26). Recent studies have demonstrated NNK exerts lung tumor-specific genotoxic effects by acting as an agonist for the α7 nicotinic acetylcholine receptor, which regulates downstream activation of signaling networks involved in the promotion and progression of lung cancer, including the PI3K/AKT and MAPK/ERK pathways (37, 38). NNK has also been shown to activate the β_2_-adrenergic receptor, which transactivates the EGFR and Raf-1/ERK1/2 signaling cascades, both of which are also well characterized for their roles in lung cancer progression and metastasis (39, 40). We found that NNK exposure was not sufficient to mimic the known human tobacco-associated genetic signatures in lung cancer (i.e., Signatures 4 and 5, ref. 22), which are likely dominated by benzopyrene, but was highly reflective of an alkylating signature consistent with the chemical attack of NNK on DNA (Signature 11) and induced the most frequent tobacco-associated alterations, including *Kras* and *Trp53*. This is not surprising as the complex mixture found in tobacco smoke may initiate other mutational processes beyond that of NNK.

FVBW-17 cells grew reproducibly and aggressively in FVB/N mice, resulting in spontaneous metastasis to multiple sites. Although ICIs have revolutionized treatment of patients with NSCLC, approximately 80% of patients do not respond (2, 3). A key hurdle to advancement of immune-based treatments is the availability of immune-competent preclinical models that recapitulate human disease. Although we found a relatively low immune infiltrate in the s.c. tumors at a late time point, immune infiltrate at earlier time points or in the lung following orthotopic implantation has not yet been evaluated. Further comprehensive immunophenotyping is also warranted as well as a direct head-to-head comparison with other syngeneic lines, including LLC. It is also possible that this model may have immune-suppressive myeloid-derived suppressor cells or macrophages in the TME. Based on our preliminary finding of anti–PD-L1 activity, this model will be useful to interrogate whether blocking the interaction of PD-1 and PD-L1 could cause tumor regression and block metastasis. Because only a subset of tumors responded to anti–PD-L1, tumor inhibition may require a multipronged approach of combinations of treatments to facilitate immune recruitment and activation of an antitumor response.

Overall, our findings show that the tobacco carcinogen–induced FVBW-17 LUAD murine cell line shares genomic and histological similarity to human LUAD, grows reproducibly in FVB/N mice, and metastasizes to distant sites. This tobacco carcinogen–induced syngeneic model of LUAD with PD-L1 expression and high TMB is a major technical advance that will allow impactful preclinical experimental studies.

## Methods

### Generation and characterization of the FVBW-17 cell line.

LUADs were induced by treating a 6-week-old female FVB/N mouse (The Jackson Laboratory) with NNK (Toronto Research Chemicals; 120 mg/kg; 15 mg/mL solution in 0.9% NaCl) i.p. injections 3 times per week for 5 weeks starting at week 0. At week 25, the mouse was sacrificed; tumors were dissected from the lung and washed 2 times with DMEM complete medium (supplemented with 10% FBS and 1% antibiotic/antimycotic). Fresh lung tumor tissue was finely minced with scalpels and added to 24-well plates containing either DMEM complete medium or 50% DMEM complete medium supplemented with 5% sterile filtered conditioned medium (CM) from 3 bone marrow– or robustly growing osteosarcoma-derived cell lines, including MG-63 (ATCC CRL-1427), HS-5 (ATCC CRL-11882), and HS-27A (ATCC CRL-2496) cells (48–72 hours of exposure)/50% Ham’s F12 + 1% FBS and 1% antibiotic/antimycotic. We had shown that the selected marrow lines, HS-5 and HS-27A (41), express mesenchymal stem cell (MSC) markers (ref. 42 and data not shown), and CM from MSCs has been shown to augment growth of human lung cancer cell lines (43). We found that addition of CM from any of these sources to our DMEM culture medium augmented the growth of the FVBW-17 cells, with MG-63 CM increasing the growth most robustly. Once the cells were expanded, we were able to decrease the CM percentage to 1% or use DMEM complete growth medium alone with maintenance of the growth rate.

Following tissue attachment, cells were monitored for colony formation followed by cell expansion. A total of 2.5 × 10^5^ cells in PBS were injected s.c. into the right hind flank of a female FVB/N mouse for selecting transplantable cells. The established tumor was excised when it reached a size of approximately 250 mm^3^ and cultured as for the primary culture. The parental line and 3 single-cell clonal cell lines, B10, B11, and F8, were established by serial dilution in 96-well plates, followed by subculture and expansion in larger vessels. The morphology of cells was observed by phase-contrast microscopy; all images were taken on a Leica DMI3000B inverted microscope. Mouse STR profiling was performed by ATCC on the parental and clonal lines at passage 3. Cell growth properties were assessed every 24 hours over a 7-day period by counting the number of viable cells with a hemocytometer (Hausser Scientific). Basal PD-L1 expression in FVBW-17 cells was assessed by flow cytometry after staining the cells with anti-mouse PD-L1 antibody (clone 10F.9G2) APC or viability dye (BioLegend) without PD-L1 antibody (control). The fluorescence minus one technique was used to set gating controls. Cell lines were routinely tested to be mycoplasma free.

### WES.

DNA from FVBW-17 parental and clonal cell lines was extracted using QIAamp DNA Mini Kit (Qiagen). For sample preparation, 1 μg genomic DNA per sample was used as input. Sequencing libraries were prepared using Agilent SureSelectXT Mouse All Exon kit (Agilent Technologies). Briefly, fragmentation was carried out using the hydrodynamic shearing system (Covaris) to generate 180–280 bp fragments, and remaining overhangs were converted into blunt ends. After adenylation of the 3′ ends, adapter oligonucleotides were ligated and selectively enriched in a PCR. The libraries were then hybridized with a biotin-labeled probe, and magnetic beads with streptomycin were used to capture exons. Captured libraries were enriched in a PCR to add index tags to prepare for hybridization. Products were purified using the AMPure XP system (Beckman Coulter) and quantified using the Agilent Bioanalyzer 2100 System.

### Somatic variant identification.

The raw sequencing data were analyzed following protocols similar to our previous work (44) but customized to the mouse genome and the mouse strain in our study, FVB/N. The quality of raw reads was assessed by FastQC (ref. 45, v0.11.7). Paired-end reads were mapped to mouse reference genome (GRCm38) using BWA-MEM (ref. 46, v0.7.17) with a minimal requirement of mapping quality 30 and soft-clipping option activated by default. Postalignment processing included duplicate removal using Sambamba (ref. 47, v0.6.8), insertions/deletions realignment, and base quality score recalibration (BQSR) using GATK4 (ref. 48, v4.1.7.0). Since matched normal tissue was not available for sequencing, we detected putative somatic mutations by MuTect2 (ref. 49, v4.1.7.0) using the tumor-only mode with vendor-provided target BED file. MuTect2 implements smarter handling of overlapping read pairs to account for PCR error and VAF calculation in this version; hence, no prior read merging was required. Variants were further filtered by MuTect2 intrinsic filters, read orientation artifact filter, and strain-specific polymorphism filter. Variants that passed all filters were annotated using ANNOVAR (ref. 50, release November 27, 2019). Our mouse strain was FVB/N, and we used C57BL/6J (GRCm38) as the reference for read alignment and variant calling, followed by proper measures to eliminate variants identified as strain-specific polymorphisms, with the following considerations: (a) the exome capture kit was designed based on the C57BL/6J genome, with the target BED file in C57BL/6J genomic coordinates, and for our analysis, we lifted over the GRCm37 (mm9) coordinates provided in the BED file to GRCm38 (mm10) using UCSC chain files (http://hgdownload.cse.ucsc.edu/goldenpath/mm9/liftOver/); (b) the C57BL/6J gene annotation is the most comprehensive and well curated; (c) most published studies are using the C57BL/6J reference genome, and our variants reported in C57BL/6J genome coordinates will be more relevant in this context; and (d) we took steps to account for the strain-specific differences in alignment refinement and variant filtering, including using FVB/N dbSNP (v142) (Sanger MGI) as the known sites of variation for postalignment BQSR and removing all variants present in FVB/N dbSNP (v142) after the variant calling. The summary of variant filtering statistics is provided in Supplemental Table 2.

### Mutation signature analysis.

The TMB was calculated by the total number of mutations that were predicted to cause a protein sequence change (a.k.a. NSSMs), including nonsynonymous, stop-gain, and stop-loss single nucleotide variants; frameshift and non-frameshift insertions/deletions (indels); and variants that modify splicing sites. For mutation signature analysis, the trinucleotide sequence context of each variant was retrieved from the mouse genome using *genCountMatrixFromVcf* from Bioconductor package signer (v1.10.0) with object *BSgenome.Mmusculus.UCSC.mm10*. The mutation lollipop figures were generated using MutationMapper from cBioPortal (https://www.cbioportal.org/mutation_mapper) (accessed September 22, 2020). *Kras* mutation was validated by Sanger sequencing.

### TCGA LUAD analysis.

The level 2 open-access somatic mutation data and clinical tables were downloaded from Broad Institute’s GDAC Firehose portal (https://gdac.broadinstitute.org/) (release March 18, 2016) for primary tumors from 512 LUAD patients in TCGA. The mutation annotation format file was imported using maftools (ref. 51, v2.0.16), and TMB was calculated as the total number of NSSMs in tumor. The tobacco smoking history group of TCGA LUAD patients was retrieved from the clinical table and coded in 1 to 5. We were unable to locate documentation of the codes; therefore we attempted to decipher codes 1 to 5 as follows: code 1 to 4 was deciphered by matching the column “tobacco_smoking_history” in the clinical table from GDAC to “Smoking.status” column in a table from the TCGA LUAD study (Supplementary Table 7 in ref. 52), and code 5 was deciphered by community knowledge (https://groups.google.com/g/cbioportal/c/irEXZRj9Who?pli=1) with reference to National Cancer Institute (NCI) CDE Browser. As a result, codes 1 to 5 were defined as: 1 = lifelong nonsmoker, 2 = current smoker, 3 = current reformed smoker for > 15 years, 4 = current reformed smoker for ≤ 15 years, and 5 = current reformed smoker, duration not specified. Patients with missing tobacco smoking history (*n* = 14) or code 5 (*n* = 4) were excluded, leaving a total of 494 patients for further analysis. Among those, 471 had exome data available and hence were included for TMB calculation.

### Evaluation of in vivo tumor growth.

FVBW-17 cells were grown to subconfluent states, trypsinized, dispersed in 1× PBS, and injected s.c. (12.5 × 10^5^ to 7.5 × 10^5^ cells in 100 μL PBS), i.v. (5 × 10^5^ cells in 100 μL PBS), or directly into the parenchyma of the left lung lobe through the rib cage using a 30G needle (1 × 10^6^ cells in 40 μL PBS containing Matrigel at final concentration 1.35 mg/mL) into 6- to 8-week-old FVB/N mice (The Jackson Laboratory) (both male and female mice). For the orthotopic injection, surgery was performed under inhaled isoflurane anesthesia, and the incision was closed using 3M VetBond adhesive. Mice were followed for up to 4 weeks. Flank tumors were measured twice weekly with digital calipers, and tumor volume was calculated as length × width^2^/2. After 21 days, s.c. tumors were removed and fixed in 10% formalin for histologic evaluation. For metastasis studies, the s.c. tumors were surgically excised under isoflurane anesthesia, and the excision wound was closed with 3M VetBond tissue adhesive. Mice were monitored for distant metastases up to 2 additional months. For i.v. and orthotopic models, lungs were formalin inflated via a cannula inserted through the trachea under 25 cm intra-alveolar pressure. FFPE tumor tissues from flank tumors, lungs, or other tissues were prepared and sliced into 5 μm sections for histologic assessment. Tumor morphology was evaluated by H&E staining and analyzed by a board-certified thoracic pathologist. Bright-field images were captured using a Leica DMI3000B microscope and Leica Application Suite v4.7 software.

### Tumor infiltrating immune profiling.

In a separate group of mice, tumors (s.c.) and spleens were processed for flow cytometric analysis of T cell populations. Excised tumors were injected with 1.5 mL of digestion solution (50 μg/mL Liberase DL research grade from Roche and 10 U/mL DNase I from MilliporeSigma in RPMI), incubated 3 minutes at room temperature, chopped into small pieces, and incubated in a total volume of 5 mL digestion solution for 15 minutes at 37°C. Digested tumor pieces and spleens were mechanically dissociated between frosted glass slides and filtered through 70 μm cell strainers (Corning) to generate single-cell suspensions. Red blood cells were lysed with 1 mL of 150 mM NH_4_Cl, 10 mM NaHCO_3_, and 0.1 mM EDTA at room temperature, and lysis was quenched with 4 mL RPMI after 30 seconds (spleen) or immediately (tumors). Cell suspensions were then counted, and 2 × 10^6^ cells were stained as follows: 15 minutes at 4°C in FCS buffer (2% FBS in PBS) with antibodies against surface antigens (CD45, clone 30-F11; CD8, clone 53-6.7; CD4, clone GK1.5; all antibodies were purchased from eBiosciences, Thermo Fisher Scientific), 10 minutes at 4°C in PBS with eFluor780 viability dye, 15 minutes at room temperature in Fixation/Permeabilization reagent (eBioscience, Thermo Fisher Scientific), and 45 minutes at room temperature in 1× Permeabilization Buffer (eBioscience, Thermo Fisher Scientific) with antibody against nuclear Foxp3 (eBioscience, Thermo Fisher Scientific, clone FJK-16S). Cells were washed once in FCS buffer prior to surface staining, washed twice in FSC buffer before viability dye staining and fixation, washed twice in 1× Permeabilization Buffer before and after nuclear staining, and washed once in FSC buffer as a final wash before acquisition. Uncompensated data were collected using FACSDiva software and LSRFortessa cytometer (BD Biosciences). Compensation and analyses were performed using FlowJo v10 software. Single-color-stained and unstained spleen cells were stained for single-color compensation controls.

### Generation of GFP-Luc–expressing cells.

FVBW-17 cells were stably transfected with lentiviral vector encoding GFP and luciferase (LL310PA-1; System Biosciences) using polybrene according to the manufacturer’s instructions. Briefly, 1 × 10^6^ FVBW-17 cells were seeded in a T25 flask. The next day, the transfection mixture of 4 mL of media, 4 μL of polybrene (8 mg/mL), and 1 mL of virus was added to the cells and incubated overnight. After 24 hours, the medium was replaced with fresh medium (without virus and polybrene) for an additional 48 hours. After 48 hours, 1 μg/mL puromycin was added to the medium. The cells were trypsinized and cultured in T75 flasks for 10 days in growth media containing 1 μg/mL puromycin, with fresh media added every other day. After 10 days, puromycin was increased to 2.5 μg/mL, and the cells were grown for an additional 10–15 days. Luciferase expression from stably transfected cells was confirmed by imaging (IVIS Lumina XR System, PerkinElmer) of 10,000–80,000 cells seeded in a 12-well plate compared with cells without the GFP-Luc gene and luciferin alone. GFP expression was confirmed by fluorescence microscopy and flow cytometry. In vivo experiments were performed with stable luciferase and GFP-transfected cells cultured in media containing 0.5 μg/mL puromycin. For in vivo bioluminescence imaging, the mice were injected with d-luciferin potassium salt (150 mg/kg, i.p.), anesthetized with isoflurane (3% for induction; 1.5% for maintenance), and imaged. Tissues were removed and imaged ex vivo for evidence of metastatic spread. Analysis was done using the Living Imaging software (PerkinElmer; version 4.3.1).

### PD-L1 antibody blockade.

Monoclonal antibody against mouse PD-L1 and mouse IgG1 control antibody were provided by AstraZeneca through a material transfer agreement. Tumor-bearing mice were injected i.p. 2 times/wk at a dose of 10 mg/kg.

### Availability of data and materials.

Raw sequencing data were uploaded to the public Sequence Read Archive database (https://www.ncbi.nlm.nih.gov/sra) in the National Center for Biotechnology Information (accession code PRJNA670224). We will send this cell line to anyone immediately upon completion of a material transfer agreement with the University of Pittsburgh.

### Study approval.

All procedures in mice were reviewed and approved by the University of Pittsburgh Institutional Animal Care and Use Committee and performed in accordance with relevant guidelines for tumor burden in mice.

### Statistics.

A 2-tailed Student’s *t* test was used for the anti–PD-L1 experiments. A *P* value of less than 0.05 was considered significant. Prism 7.0 (GraphPad) was used for all analyses.

## Author contributions

LPS conducted experiments, was responsible for the overall experimental design, data analysis and interpretation, and wrote the manuscript; AGD established the cell lines and coordinated WES and STR profiling; EHH and PD performed in vivo experiments; VK established the GFP-Luc cells; FPV performed flow cytometry and data analysis; SD provided all histologic analysis; RB performed bioinformatics WES analysis and manuscript preparation; RAS provided intellectual input and provided conditioned medium; TFB oversaw the experimental design and data analysis and contributed to manuscript preparation; and CJB provided intellectual input, oversaw the experimental design and data analysis, and contributed to manuscript preparation.

## Supplementary Material

Supplemental data

## Version 1 12/22/2020

In-Press Preview

## Version 2 02/08/2021

Electronic publication
