# Supplementary material for: Syngeneic tobacco carcinogen–induced mouse lung adenocarcinoma model exhibits PD-L1 expression and high tumor mutational burden
Source: JCI Insight. 2021 Feb 8;6(3):e145307. doi: 10.1172/jci.insight.145307 (PMC7934870; doi:10.1172/jci.insight.145307)
Supplement: Supplemental data [file jciinsight-6-145307-s037.pdf]

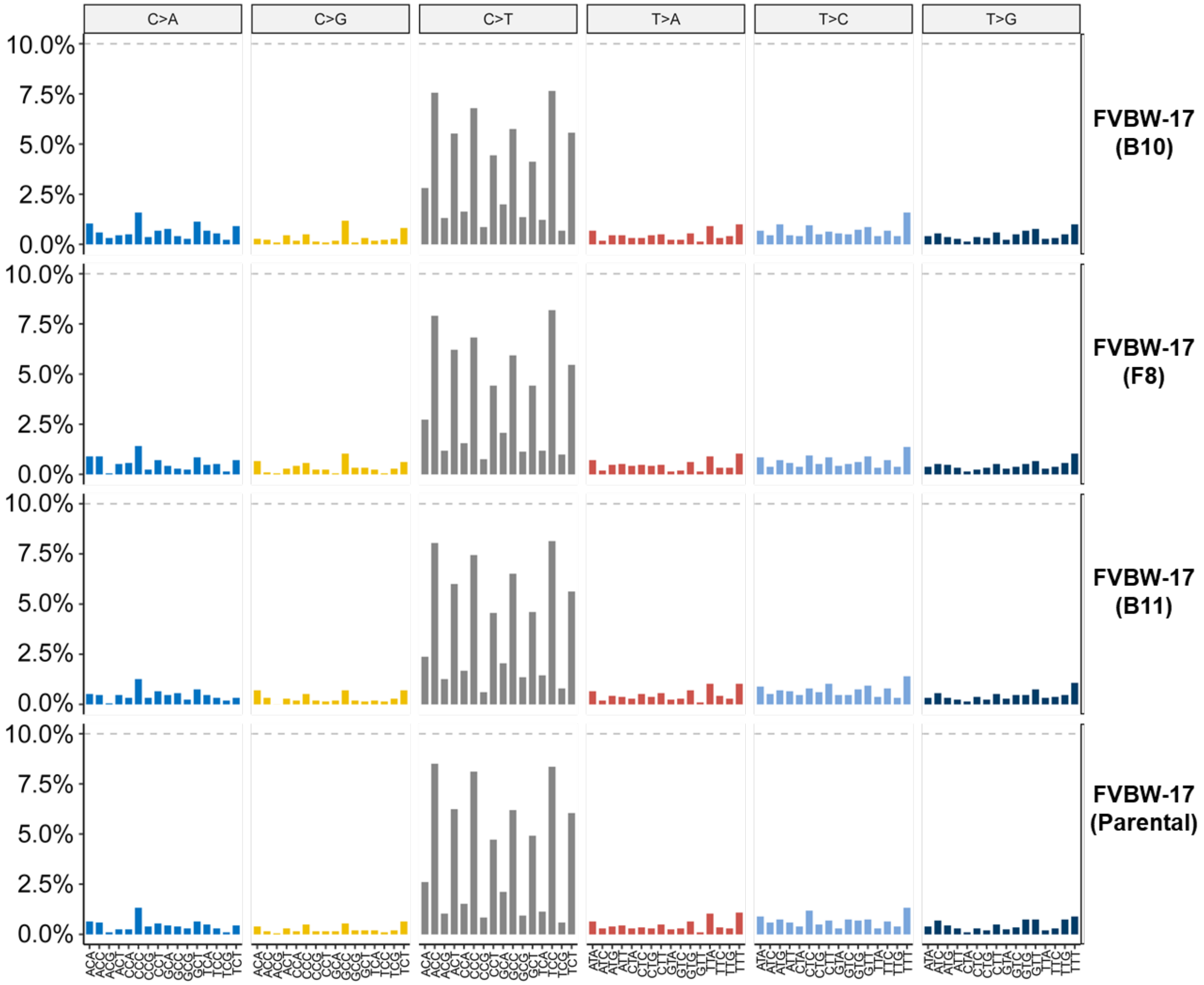

Figure S1. Mutation signature of all FVBW-17 cell lines associated with alkylating agents using a 96-substitution classification. FVBW-17 is the parental strain, and the other three are clonal cell lines.

Table S1. Number of mice and frequency of distant metastases of FVBW-17 cells from s.c. tumors.

	Lung	Spleen	Kidney	Liver
**Male (n=15)**	9 (60%)	3 (20%)	3 (20%)	2 (13%)
**Female (n=15)**	11 (73%)	4 (27%)	2 (13%)	2 (13%)

Table S2. Summary statistics of variant filtering steps.

Sample	Raw list of variants	After removing strain-specific polymorphisms (those present in FVB/NJ dbSNP v142)	Variants PASS (removing reads flagged by MuTect2 intrinsic filters in tumor-only mode or by read orientation filter)	Putative somatic variants in coding region (exonic or splicing)	Putative somatic variants predicted to affect protein sequences (a.k.a. non-synonymous somatic mutations, NSSMs)	NSSM single nucleotide variants (SNVs)	NSSM insetions/ deletions (indels)
FVBW-17	176459	22316	3307	778	506	442	64
FVBW-17 B10	177689	23040	3519	873	577	504	73
FVBW-17 F8	177203	22745	3424	832	563	495	68
FVBW-17 B11	177149	22803	3414	874	579	506	73
